# Benzimidazolium 3,5-dicarb­oxy­benzoate trihydrate

**DOI:** 10.1107/S1600536810023305

**Published:** 2010-06-23

**Authors:** Hong Chang, You-Li Yang, Xiu-Guang Wang, En-Cui Yang

**Affiliations:** a College of Chemistry, Tianjin Key Laboratory of Structure and Performance for Functional Molecule, Tianjin Normal University, Tianjin 300387, People’s Republic of China

## Abstract

Cocrystallization of benzimidazole with benzene 1,3,5-tricarb­oxy­lic acid in slightly basic medium afforded the title compound, C_7_H_7_N_2_^+^·C_9_H_5_O_6_^−^·3H_2_O, in which one of the imidazole N atom is protonated and one carb­oxy­lic group of aromatic acid is deprotonated. In the crystal structure, inter­molecular N—H⋯O hydrogen-bonding connects the two organic components into dimers, which are further linked into a three-dimensional network by O—H⋯O and N—H⋯O inter­actions between the water mol­ecules and the dimers.

## Related literature

For mol­ecular self-assembly by non-covalent inter­actions and its potential applications, see: Remenar *et al.* (2003 ▶); Oxtoby *et al.* (2005 ▶); Zaworotko (2001 ▶). For the benzimidazole-based supra­molecular aggregate formed by π–π stacking and hydrogen-bonding inter­actions, see Gao *et al.* (2004 ▶).
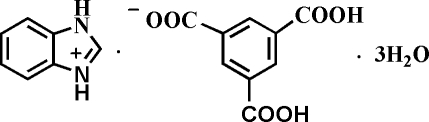


## Experimental

### 

#### Crystal data

C_7_H_7_N_2_^+^·C_9_H_5_O_6_^−^·3H_2_O
*M* *_r_* = 382.32
Triclinic, 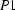

*a* = 3.8478 (2) Å
*b* = 10.2231 (6) Å
*c* = 11.2982 (7) Å
α = 85.522 (1)°
β = 80.707 (1)°
γ = 81.826 (1)°
*V* = 433.45 (4) Å^3^
*Z* = 1
Mo *K*α radiation
μ = 0.12 mm^−1^
*T* = 296 K
0.24 × 0.22 × 0.20 mm

#### Data collection

Bruker APEXII CCD area-detector diffractometer
Absorption correction: multi-scan (*SADABS*; Sheldrick, 1996 ▶) *T*_min_ = 0.971, *T*_max_ = 0.976
2240 measured reflections
1536 independent reflections
1478 reflections with *I* > 2σ(*I*)
*R*_int_ = 0.008

#### Refinement

*R*[*F* ^2^ > 2σ(*F* ^2^)] = 0.028
*wR*(*F* ^2^) = 0.073
*S* = 1.04
1536 reflections
246 parameters
3 restraints
H-atom parameters constrained
Δρ_max_ = 0.14 e Å^−3^
Δρ_min_ = −0.19 e Å^−3^

### 

Data collection: *APEX2* (Bruker, 2003 ▶); cell refinement: *SAINT* (Bruker, 2001 ▶); data reduction: *SAINT*; program(s) used to solve structure: *SHELXS97* (Sheldrick, 2008 ▶); program(s) used to refine structure: *SHELXL97* (Sheldrick, 2008 ▶); molecular graphics: *SHELXTL* (Sheldrick, 2008 ▶) and *DIAMOND* (Brandenburg & Berndt, 1999 ▶); software used to prepare material for publication: *SHELXL97*.

## Supplementary Material

Crystal structure: contains datablocks I, global. DOI: 10.1107/S1600536810023305/nc2189sup1.cif

Structure factors: contains datablocks I. DOI: 10.1107/S1600536810023305/nc2189Isup2.hkl

Additional supplementary materials:  crystallographic information; 3D view; checkCIF report

## Figures and Tables

**Table 1:** Hydrogen-bond geometry (Å, °)

*D*—H⋯*A*	*D*—H	H⋯*A*	*D*⋯*A*	*D*—H⋯*A*
O4—H4⋯O9^i^	0.82	1.73	2.539 (2)	169
O6—H6⋯O7^ii^	0.82	1.77	2.589 (2)	176
N1—H1⋯O8^iii^	0.86	1.97	2.812 (3)	167
N2—H2⋯O2^iv^	0.86	1.87	2.721 (3)	173
O7—H7*A*⋯O2	0.85	1.92	2.760 (3)	173
O7—H7*B*⋯O8	0.85	2.09	2.877 (3)	155
O8—H8*A*⋯O5^v^	0.85	1.99	2.797 (3)	159
O8—H8*B*⋯O3^iii^	0.85	1.92	2.730 (2)	160
O9—H9*A*⋯O1^iv^	0.85	1.81	2.654 (2)	173
O9—H9*B*⋯O1^vi^	0.85	1.86	2.685 (3)	163

Symmetry codes: (i) 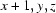; (ii) 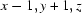; (iii) 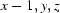; (iv) 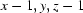; (v) 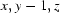; (vi) 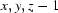.

**Fig. 1.**
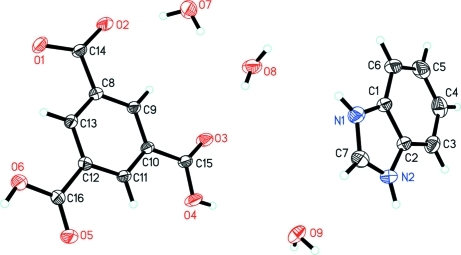
The molecular structure of the title compound with labeling and displacement ellipsoids drawn at the 30% probability level.

**Fig. 2.**
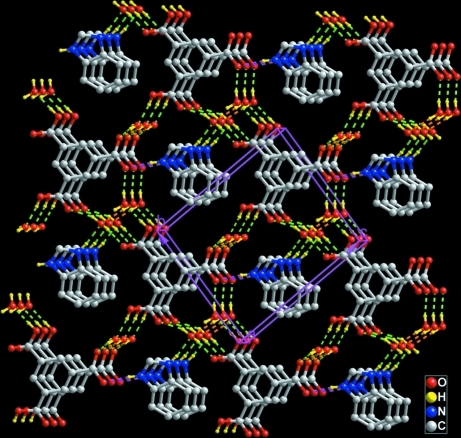
Crystal structure of the title compound with view along the a-axis. Hydrogen bonding is shown as dashed lines.

## supplementary crystallographic information

### Comment

Recently, molecular self-assembly by non-covalent interactions has attacted considerable interest in supramolecular chemistry and crystal engineering due to their possible potential applications as functional materials (Zaworotko, 2001), in molecular recognition (Oxtoby *et al.*, 2005), and pharmaceutical chemistry (Remenar *et al., *2003). Obviously, the conguated organic components with rich carboxylate or amino groups have became good blocks for the construction of self-assembly systems, since hydrogen-bonding and π··· π interactions are the main driven forces of the assembly process. In this context benzimidazole (bim) and its derivatives have been used as promising building blocks for the construction of supramolecular aggregates. As a continuation of this work bim and 1,3,5-benzenetricarboxylic acid (H_3_btc) were reacted in slightly basic medium and the product were identified by single crystal X-ray diffraction.

The asymmetric unit of the title compound comprises one Hbim^+^ cation, one monodeprotonated H_2_btc^-^ anion and three water molecules all of the located in general positions (Fig. 1). The two carboxyl groups are located within the plane of the aromatic ring, whereas the deprotonated carboxylate group is slightly twisted out of the ring plane. The dihedral angle between the aromatic and the benzimidazole rings amount to 6.84 (5)*^o^*.

In the crystal structure, the Hbim^+^ cations and H_2_btc^-^ anions are connected into dimers by N–H···O hydrogen-bonding between the N—H H atoms and the carboxylate group (Table 1). These dimers are further connected by O–H···O and N–H···O hydrogen bonding between the water molecules, the carboxyl and carboxylate groups as well as the N-H H atoms into a three-dimensional hydrogen bonded network (Figure 2 and Table 1). In this interactions the water molecules act as hydrogen bond donor and acceptor.

### Experimental

A mixture containing H_3_btc (21.0 mg, 0.1 mmol), bim (11.8 mg, 0.1 mmol), and NaOH (4.0 mg, 0.1 mmol) was dissolved in a mixed methanol-H_2_O solution (v: v = 1:1, 10.0 ml). Then, the mixture was transferred into a Teflon-lined reactor (23.0 ml) and heated to 70 *^o^*C for 48 hrs. After the mixture was cooled to room temperature at a rate of 6 *^o^*C h^-1^, colorless block-shaped crystals suitable for X-ray diffraction were obtained directly. Yield: 56% based on bim. Anal.Calcd for C_16_H_18_N_2_O_9_,*C*,50.26; H, 4.75; N, 7.33%. Found: C, 50.32; H, 4.78; N,7.36%.

### Refinement

The C-H, N-H and hydroxy H atoms were located in difference maps, but were placed in calculated positions (O-H allowed to rotate but not to tip) and treated as riding, with C – H = 0.93, O – H = 0.82 and N – H = 0.86 Å. The water H atoms were located in difference map and were refined using restraints. All H were refined isotropic with [*U*_iso_(H) = 1.2 *U*_eq_(C, N) or 1.5 *U*_eq_(O)].

### Crystal data

**Table:**

C_7_H_7_N_2_^+^·C_9_H_5_O_6_^−^·3H_2_O	*Z* = 1
*M**_r_* = 382.32	*F*(000) = 200
Triclinic, *P*1	*D*_x_ = 1.465 Mg m^−^^3^
Hall symbol: P1	Mo *K*α radiation, λ = 0.71073 Å
*a* = 3.8478 (2) Å	Cell parameters from 1795 reflections
*b* = 10.2231 (6) Å	θ = 2.8–27.9°
*c* = 11.2982 (7) Å	µ = 0.12 mm^−^^1^
α = 85.522 (1)°	*T* = 296 K
β = 80.707 (1)°	Block, colourless
γ = 81.826 (1)°	0.24 × 0.22 × 0.20 mm
*V* = 433.45 (4) Å^3^	

### Data collection

**Table:**

Bruker APEXII CCD area-detector diffractometer	1536 independent reflections
Radiation source: fine-focus sealed tube	1478 reflections with *I* > 2σ(*I*)
graphite	*R*_int_ = 0.008
phi and ω scans	θ_max_ = 25.0°, θ_min_ = 2.0°
Absorption correction: multi-scan (*SADABS*; Sheldrick, 1996)	*h* = −4→4
*T*_min_ = 0.971, *T*_max_ = 0.976	*k* = −12→5
2240 measured reflections	*l* = −13→13

### Refinement

**Table:**

Refinement on *F*^2^	Primary atom site location: structure-invariant direct methods
Least-squares matrix: full	Secondary atom site location: difference Fourier map
*R*[*F*^2^ > 2σ(*F*^2^)] = 0.028	Hydrogen site location: inferred from neighbouring sites
*wR*(*F*^2^) = 0.073	H-atom parameters constrained
*S* = 1.04	*w* = 1/[σ^2^(*F*_o_^2^) + (0.0522*P*)^2^ + 0.0236*P*] where *P* = (*F*_o_^2^ + 2*F*_c_^2^)/3
1536 reflections	(Δ/σ)_max_ < 0.001
246 parameters	Δρ_max_ = 0.14 e Å^−^^3^
3 restraints	Δρ_min_ = −0.18 e Å^−^^3^

### Special details

**Table:**

Geometry. All e.s.d.'s (except the e.s.d. in the dihedral angle between two l.s. planes) are estimated using the full covariance matrix. The cell e.s.d.'s are taken into account individually in the estimation of e.s.d.'s in distances, angles and torsion angles; correlations between e.s.d.'s in cell parameters are only used when they are defined by crystal symmetry. An approximate (isotropic) treatment of cell e.s.d.'s is used for estimating e.s.d.'s involving l.s. planes.
Refinement. Refinement of *F*^2^ against ALL reflections. The weighted *R*-factor *wR* and goodness of fit *S* are based on *F*^2^, conventional *R*-factors *R* are based on *F*, with *F* set to zero for negative *F*^2^. The threshold expression of *F*^2^ > σ(*F*^2^) is used only for calculating *R*-factors(gt) *etc*. and is not relevant to the choice of reflections for refinement. *R*-factors based on *F*^2^ are statistically about twice as large as those based on *F*, and *R*- factors based on ALL data will be even larger.

### Fractional atomic coordinates and isotropic or equivalent isotropic displacement parameters (Å^2^)

**Table:**

	*x*	*y*	*z*	*U*_iso_*/*U*_eq_	
O1	0.8287 (5)	0.63795 (18)	1.20720 (14)	0.0482 (4)	
O2	0.9531 (6)	0.44710 (18)	1.11874 (15)	0.0581 (5)	
O3	1.2332 (6)	0.50067 (17)	0.65819 (16)	0.0552 (5)	
O4	0.9893 (5)	0.68726 (18)	0.57532 (15)	0.0546 (5)	
H4	1.1005	0.6561	0.5135	0.082*	
O5	0.2872 (6)	1.03786 (17)	0.80326 (17)	0.0596 (5)	
O6	0.1819 (5)	1.01258 (17)	1.00192 (16)	0.0544 (5)	
H6	0.0662	1.0858	0.9946	0.082*	
N1	−0.0881 (6)	0.2463 (2)	0.52127 (19)	0.0457 (5)	
H1	−0.1644	0.2470	0.5971	0.055*	
N2	0.0014 (6)	0.3109 (2)	0.33346 (19)	0.0458 (5)	
H2	−0.0077	0.3595	0.2681	0.055*	
C1	0.1306 (6)	0.1435 (2)	0.4635 (2)	0.0384 (5)	
C2	0.1892 (6)	0.1855 (2)	0.3429 (2)	0.0387 (5)	
C3	0.3998 (7)	0.1058 (3)	0.2576 (2)	0.0497 (6)	
H3	0.4411	0.1336	0.1769	0.060*	
C4	0.5439 (7)	−0.0170 (3)	0.2996 (3)	0.0587 (7)	
H4A	0.6863	−0.0735	0.2455	0.070*	
C5	0.4822 (8)	−0.0587 (3)	0.4207 (3)	0.0592 (8)	
H5	0.5843	−0.1424	0.4450	0.071*	
C6	0.2766 (7)	0.0196 (3)	0.5047 (3)	0.0507 (6)	
H6A	0.2364	−0.0086	0.5854	0.061*	
C7	−0.1607 (7)	0.3433 (2)	0.4406 (2)	0.0482 (6)	
H7	−0.3052	0.4227	0.4574	0.058*	
C8	0.7899 (5)	0.6418 (2)	1.00125 (19)	0.0329 (5)	
C9	0.9324 (5)	0.5896 (2)	0.89162 (19)	0.0325 (4)	
H9	1.0628	0.5057	0.8895	0.039*	
C10	0.8824 (6)	0.6614 (2)	0.78491 (19)	0.0320 (4)	
C11	0.6801 (5)	0.7855 (2)	0.78785 (19)	0.0332 (4)	
H11	0.6451	0.8336	0.7165	0.040*	
C12	0.5300 (6)	0.8377 (2)	0.8973 (2)	0.0339 (5)	
C13	0.5874 (5)	0.7666 (2)	1.00353 (19)	0.0336 (4)	
H13	0.4907	0.8023	1.0768	0.040*	
C14	0.8615 (6)	0.5697 (2)	1.11777 (19)	0.0367 (5)	
C15	1.0532 (6)	0.6071 (2)	0.66716 (18)	0.0358 (5)	
C16	0.3216 (6)	0.9717 (2)	0.8957 (2)	0.0388 (5)	
O7	0.8307 (6)	0.24730 (19)	0.9861 (2)	0.0668 (6)	
H7A	0.8766	0.3119	1.0213	0.100*	
H7B	0.7602	0.2814	0.9216	0.100*	
O8	0.5721 (6)	0.27703 (19)	0.75910 (17)	0.0584 (5)	
H8A	0.4356	0.2174	0.7739	0.088*	
H8B	0.4373	0.3479	0.7446	0.088*	
O9	0.2592 (5)	0.60054 (19)	0.37164 (15)	0.0520 (5)	
H9A	0.1063	0.6114	0.3240	0.078*	
H9B	0.4479	0.6232	0.3300	0.078*	

### Atomic displacement parameters (Å^2^)

**Table:**

	*U*^11^	*U*^22^	*U*^33^	*U*^12^	*U*^13^	*U*^23^
O1	0.0596 (10)	0.0565 (10)	0.0271 (8)	0.0005 (8)	−0.0094 (7)	−0.0032 (7)
O2	0.0961 (15)	0.0393 (9)	0.0363 (9)	0.0075 (9)	−0.0217 (9)	0.0072 (7)
O3	0.0797 (12)	0.0418 (10)	0.0325 (9)	0.0211 (9)	−0.0007 (8)	0.0009 (7)
O4	0.0773 (12)	0.0496 (10)	0.0247 (8)	0.0198 (9)	0.0002 (8)	0.0047 (7)
O5	0.0951 (15)	0.0369 (9)	0.0398 (10)	0.0166 (9)	−0.0136 (9)	0.0021 (8)
O6	0.0812 (13)	0.0359 (9)	0.0385 (9)	0.0153 (8)	−0.0060 (9)	−0.0032 (7)
N1	0.0534 (11)	0.0466 (12)	0.0355 (11)	−0.0055 (9)	−0.0028 (9)	−0.0024 (9)
N2	0.0611 (13)	0.0366 (10)	0.0406 (11)	−0.0060 (9)	−0.0160 (10)	0.0087 (8)
C1	0.0440 (11)	0.0384 (11)	0.0346 (11)	−0.0078 (9)	−0.0119 (9)	0.0034 (9)
C2	0.0462 (12)	0.0371 (12)	0.0355 (11)	−0.0099 (10)	−0.0131 (10)	0.0036 (9)
C3	0.0536 (14)	0.0597 (16)	0.0371 (13)	−0.0118 (12)	−0.0048 (11)	−0.0075 (12)
C4	0.0533 (15)	0.0527 (16)	0.072 (2)	−0.0013 (12)	−0.0138 (14)	−0.0202 (14)
C5	0.0596 (16)	0.0382 (13)	0.082 (2)	0.0024 (11)	−0.0281 (15)	0.0001 (14)
C6	0.0617 (15)	0.0440 (14)	0.0493 (14)	−0.0071 (12)	−0.0229 (12)	0.0107 (11)
C7	0.0556 (14)	0.0381 (13)	0.0503 (15)	−0.0012 (11)	−0.0105 (12)	−0.0021 (11)
C8	0.0370 (11)	0.0335 (11)	0.0276 (10)	−0.0049 (9)	−0.0052 (8)	0.0024 (8)
C9	0.0373 (11)	0.0297 (10)	0.0282 (10)	0.0016 (8)	−0.0046 (8)	0.0012 (8)
C10	0.0376 (10)	0.0315 (10)	0.0254 (10)	−0.0016 (8)	−0.0046 (8)	0.0009 (8)
C11	0.0397 (10)	0.0307 (10)	0.0274 (11)	−0.0001 (8)	−0.0064 (8)	0.0032 (8)
C12	0.0388 (10)	0.0297 (10)	0.0337 (12)	−0.0030 (8)	−0.0085 (9)	−0.0006 (9)
C13	0.0394 (11)	0.0321 (11)	0.0280 (10)	−0.0007 (9)	−0.0040 (8)	−0.0028 (8)
C14	0.0421 (11)	0.0389 (12)	0.0267 (11)	0.0014 (9)	−0.0061 (9)	0.0025 (9)
C15	0.0412 (11)	0.0350 (12)	0.0281 (11)	0.0024 (9)	−0.0038 (9)	0.0021 (9)
C16	0.0485 (12)	0.0308 (11)	0.0363 (12)	0.0012 (9)	−0.0093 (10)	−0.0037 (10)
O7	0.0998 (16)	0.0385 (9)	0.0595 (12)	0.0173 (9)	−0.0252 (11)	−0.0076 (9)
O8	0.0766 (13)	0.0444 (10)	0.0464 (10)	0.0072 (8)	−0.0049 (9)	0.0089 (8)
O9	0.0559 (10)	0.0711 (12)	0.0274 (8)	−0.0039 (9)	−0.0054 (7)	−0.0029 (8)

### Geometric parameters (Å, °)

**Table:**

O1—C14	1.252 (3)	C5—H5	0.9300
O2—C14	1.252 (3)	C6—H6A	0.9300
O3—C15	1.206 (3)	C7—H7	0.9300
O4—C15	1.304 (3)	C8—C9	1.386 (3)
O4—H4	0.8200	C8—C13	1.397 (3)
O5—C16	1.214 (3)	C8—C14	1.503 (3)
O6—C16	1.308 (3)	C9—C10	1.388 (3)
O6—H6	0.8200	C9—H9	0.9300
N1—C7	1.325 (3)	C10—C11	1.390 (3)
N1—C1	1.384 (3)	C10—C15	1.496 (3)
N1—H1	0.8600	C11—C12	1.390 (3)
N2—C7	1.313 (4)	C11—H11	0.9300
N2—C2	1.384 (3)	C12—C13	1.385 (3)
N2—H2	0.8600	C12—C16	1.487 (3)
C1—C6	1.388 (3)	C13—H13	0.9300
C1—C2	1.389 (3)	O7—H7A	0.8500
C2—C3	1.390 (4)	O7—H7B	0.8499
C3—C4	1.378 (4)	O8—H8A	0.8500
C3—H3	0.9300	O8—H8B	0.8500
C4—C5	1.394 (5)	O9—H9A	0.8500
C4—H4A	0.9300	O9—H9B	0.8500
C5—C6	1.366 (5)		
			
C15—O4—H4	109.5	C9—C8—C14	121.44 (18)
C16—O6—H6	109.5	C13—C8—C14	119.20 (18)
C7—N1—C1	108.8 (2)	C8—C9—C10	120.62 (18)
C7—N1—H1	125.6	C8—C9—H9	119.7
C1—N1—H1	125.6	C10—C9—H9	119.7
C7—N2—C2	108.8 (2)	C9—C10—C11	119.75 (19)
C7—N2—H2	125.6	C9—C10—C15	120.17 (18)
C2—N2—H2	125.6	C11—C10—C15	120.06 (19)
N1—C1—C6	132.4 (2)	C12—C11—C10	120.10 (19)
N1—C1—C2	105.9 (2)	C12—C11—H11	120.0
C6—C1—C2	121.7 (2)	C10—C11—H11	120.0
N2—C2—C1	106.4 (2)	C13—C12—C11	119.81 (19)
N2—C2—C3	132.0 (2)	C13—C12—C16	122.13 (19)
C1—C2—C3	121.5 (2)	C11—C12—C16	118.02 (18)
C4—C3—C2	116.2 (2)	C12—C13—C8	120.39 (19)
C4—C3—H3	121.9	C12—C13—H13	119.8
C2—C3—H3	121.9	C8—C13—H13	119.8
C3—C4—C5	121.9 (3)	O1—C14—O2	124.6 (2)
C3—C4—H4A	119.1	O1—C14—C8	116.94 (19)
C5—C4—H4A	119.1	O2—C14—C8	118.41 (19)
C6—C5—C4	122.0 (3)	O3—C15—O4	123.5 (2)
C6—C5—H5	119.0	O3—C15—C10	123.5 (2)
C4—C5—H5	119.0	O4—C15—C10	113.00 (18)
C5—C6—C1	116.6 (3)	O5—C16—O6	123.0 (2)
C5—C6—H6A	121.7	O5—C16—C12	122.6 (2)
C1—C6—H6A	121.7	O6—C16—C12	114.39 (19)
N2—C7—N1	110.0 (2)	H7A—O7—H7B	105.2
N2—C7—H7	125.0	H8A—O8—H8B	105.1
N1—C7—H7	125.0	H9A—O9—H9B	105.1
C9—C8—C13	119.31 (19)		
			
C7—N1—C1—C6	−178.5 (2)	C9—C10—C11—C12	0.3 (3)
C7—N1—C1—C2	0.8 (3)	C15—C10—C11—C12	−178.1 (2)
C7—N2—C2—C1	0.0 (3)	C10—C11—C12—C13	1.1 (3)
C7—N2—C2—C3	179.4 (2)	C10—C11—C12—C16	178.9 (2)
N1—C1—C2—N2	−0.4 (2)	C11—C12—C13—C8	−1.2 (3)
C6—C1—C2—N2	178.9 (2)	C16—C12—C13—C8	−178.9 (2)
N1—C1—C2—C3	−179.9 (2)	C9—C8—C13—C12	−0.1 (3)
C6—C1—C2—C3	−0.6 (3)	C14—C8—C13—C12	177.29 (18)
N2—C2—C3—C4	−178.9 (2)	C9—C8—C14—O1	156.9 (2)
C1—C2—C3—C4	0.4 (3)	C13—C8—C14—O1	−20.4 (3)
C2—C3—C4—C5	−0.1 (4)	C9—C8—C14—O2	−22.2 (3)
C3—C4—C5—C6	−0.2 (4)	C13—C8—C14—O2	160.5 (2)
C4—C5—C6—C1	0.1 (4)	C9—C10—C15—O3	1.2 (3)
N1—C1—C6—C5	179.4 (3)	C11—C10—C15—O3	179.6 (2)
C2—C1—C6—C5	0.3 (3)	C9—C10—C15—O4	−178.3 (2)
C2—N2—C7—N1	0.5 (3)	C11—C10—C15—O4	0.1 (3)
C1—N1—C7—N2	−0.8 (3)	C13—C12—C16—O5	175.6 (2)
C13—C8—C9—C10	1.5 (3)	C11—C12—C16—O5	−2.1 (3)
C14—C8—C9—C10	−175.82 (19)	C13—C12—C16—O6	−3.8 (3)
C8—C9—C10—C11	−1.6 (3)	C11—C12—C16—O6	178.4 (2)
C8—C9—C10—C15	176.8 (2)		

### Hydrogen-bond geometry (Å, °)

**Table:**

*D*—H···*A*	*D*—H	H···*A*	*D*···*A*	*D*—H···*A*
O4—H4···O9^i^	0.82	1.73	2.539 (2)	169
O6—H6···O7^ii^	0.82	1.77	2.589 (2)	176
N1—H1···O8^iii^	0.86	1.97	2.812 (3)	167
N2—H2···O2^iv^	0.86	1.87	2.721 (3)	173
O7—H7A···O2	0.85	1.92	2.760 (3)	173
O7—H7B···O8	0.85	2.09	2.877 (3)	155
O8—H8A···O5^v^	0.85	1.99	2.797 (3)	159
O8—H8B···O3^iii^	0.85	1.92	2.730 (2)	160
O9—H9A···O1^iv^	0.85	1.81	2.654 (2)	173
O9—H9B···O1^vi^	0.85	1.86	2.685 (3)	163

Symmetry codes: (i) *x*+1, *y*, *z*; (ii) *x*−1, *y*+1, *z*; (iii) *x*−1, *y*, *z*; (iv) *x*−1, *y*, *z*−1; (v) *x*, *y*−1, *z*; (vi) *x*, *y*, *z*−1.
